# Association of Quality and Technology With Patient Mobility for Colorectal Cancer Surgery

**DOI:** 10.1001/jamasurg.2022.5461

**Published:** 2022-11-09

**Authors:** Ajay Aggarwal, Lu Han, Jemma Boyle, Daniel Lewis, Angela Kuyruba, Michael Braun, Kate Walker, Nicola Fearnhead, Richard Sullivan, Jan van der Meulen

**Affiliations:** 1 Department of Health Services Research and Policy, London School of Hygiene and Tropical Medicine, London, United Kingdom; 2 Clinical Effectiveness Unit, Royal College of Surgeons of England, London, United Kingdom; 3 Department of Oncology, The Christie NHS Foundation Trust, Manchester, United Kingdom; 4 School of Medical Sciences, University of Manchester, United Kingdom; 5 Department of Colorectal Surgery, Cambridge University Hospitals, Cambridge, United Kingdom; 6 Institute of Cancer Policy, King’s College London, London, United Kingdom; 7 Department of Oncology, Guy’s & St Thomas’ NHS Trust, London, United Kingdom

## Abstract

**Importance:**

Many health care systems publish hospital-level quality measures as a driver of hospital performance and to support patient choice, but it is not known if patients with cancer respond to them.

**Objective:**

To investigate hospital quality and patient factors associated with treatment location.

**Design, Setting, and Participants:**

This choice modeling study used national administrative hospital data. Patients with colon and rectal cancer treated in all 163 English National Health Service (NHS) hospitals delivering colorectal cancer surgery between April 2016 and March 2019 were included. The extent to which patients chose to bypass their nearest surgery center was investigated, and conditional logistic regression was used to estimate the association of additional travel time, hospital quality measures, and patient characteristics with treatment location.

**Exposures:**

Additional travel time in minutes, hospital characteristics, and patient characteristics: age, sex, cancer T stage, socioeconomic status, comorbidity, and rural or urban residence.

**Main Outcomes and Measures:**

Treatment location.

**Results:**

Overall, 44 299 patients were included in the final cohort (mean [SD] age, 68.9 [11.6] years; 18 829 [42.5%] female). A total of 8550 of 31 258 patients with colon cancer (27.4%) and 3933 of 13 041 patients with rectal cancer (30.2%) bypassed their nearest surgical center. Travel time was strongly associated with treatment location. The association was less strong for younger, more affluent patients and those from rural areas. For rectal cancer, patients were more likely to travel to a hospital designated as a specialist colorectal cancer surgery center (odds ratio, 1.45; 95% CI, 1.13-1.87; *P* = .004) and to a hospital performing robotic surgery for rectal cancer (odds ratio, 1.43; 95% CI, 1.11-1.86; *P* = .007). Patients were less likely to travel to hospitals deemed to have inadequate care by the national quality regulator (odds ratio, 0.70; 95% CI, 0.50-0.97; *P* = .03). Patients were not more likely to travel to hospitals with better 2-year bowel cancer mortality outcomes.

**Conclusions and Relevance:**

Patients appear responsive to hospital characteristics that reflect overall hospital quality and the availability of robotic surgery but not to specific disease-related outcome measures. Policies allowing patients to choose where they have colorectal cancer surgery may not result in better outcomes but could drive inequities in the health care system.

## Key Points

**Question:**

In health care systems offering patients a choice of hospital for cancer surgery, what factors influence treatment location?

**Findings:**

In this national population-based study, patients with rectal cancer are responsive to published measures of overall hospital quality and the availability of robotic surgery but less so to cancer-related outcome measures.

**Meaning:**

Patients are responsive to hospital-level characteristics but not necessarily to those that will have an impact on their outcome.

## Teaser

This cohort study investigates hospital quality and patient factors associated with treatment location using data from the UK National Health Service.

## Introduction

Several countries have introduced policies that allow patients to choose their hospital as a means to improve quality and efficiency of health care services through price or quality competition.^1,2^ A key requirement for health care market mechanisms to function effectively is accurate and understandable measures of quality to support patient choices.

Research has demonstrated that patients with cancer are responsive to patient choice policies regardless of whether hospital-level quality indicators are available.^3^ In the UK National Health Service (NHS), 1 in 3 patients with prostate cancer undergoing surgical treatment and 1 in 5 receiving radical radiotherapy traveled beyond (ie, bypassed) their nearest center for treatment.^3,4,5,6,7,8^ In the absence of measures of treatment quality, patients with prostate cancer were attracted to centers based on surrogate indicators of quality such as the availability of robotic surgery and the reputation of hospitals.^4,6^

The attraction of patients to centers offering innovative treatments has been shown to be an incentive for technology adoption despite little evidence that they improve oncological outcomes.^8,9,10,11,12^ Patient choice policies can also have a significant impact on the equity of the system. For example, patterns of mobility have been found to be inequitable, with younger and more affluent patients prepared to travel further to receive treatment.^4,5^ It remains unclear whether these findings are generalizable to other tumor types and to what extent published patient-level outcomes following cancer treatment can act as a driver of a hospital gaining a competitive advantage.

Colorectal cancer provides an important tumor type to evaluate patient choice of hospital policies. It is a high-incidence cancer and includes 2 distinct tumor types, which are associated with differences in the technical complexity and management, which may influence decisions about where patients seek care. The NHS provides an ideal health care system for understanding the impact of patient choice policies on patterns of patient mobility and their health care system effects. National administrative data sets are available for all patients treated in the NHS (more than 95% of all cancer care is delivered in this setting). In principle, patients have no restrictions in which surgery center they choose to have treatment, but they do require a referral from a primary or secondary care physician. In addition, the NHS publishes outcome measures of overall hospital quality and the clinical quality of cancer care.^13,14^

We investigated whether patients with colorectal cancer who had a major primary resection in the NHS bypassed their nearest surgical center for treatment. We then evaluated the equity implications of patient travel patterns as well as the hospital characteristics associated with the observed mobility patterns to inform policies and incentives designed to ensure effective, efficient, and fair functioning of health care systems supporting patient choice of hospital.

## Methods

### Study Design and Setting

This is a choice modeling study undertaken in the NHS using national administrative hospital data. The study has been reported in accordance with Strengthening the Reporting of Observational Studies in Epidemiology (STROBE) reporting guideline. Ethics approval for use of secondary anonymized patient-level data sets for these analyses was received from the NHS Research Ethics Committee on June 1, 2020. Informed consent was not required for use of this information.

### Data Collection

Data were retrieved from the Hospital Episode Statistics^15^ and linked at the patient level to the National Bowel Cancer Audit records, which provided information on cancer stage.^16^ Hospital Episode Statistics provided information on each patient’s area of residence, age, sex, comorbidities, and treatment. Data on ethnicity were collected by Hospital Episode Statistics; however, there was missing data and therefore these data were not reported here. The Office for National Statistics provided information on date of death.

### Population

We obtained individual patient-level data for all patients who had been diagnosed with colorectal cancer between April 1, 2016, and March 31, 2019, and who subsequently underwent a major bowel cancer surgical resection in the NHS.

Patients were included in our analysis if they had undergone elective major resection, were treated in the 163 NHS hospitals routinely performing colorectal cancer surgery (at least 10 major resections per annum), and had nonmetastatic disease. Patients who underwent surgery in the private sector were not included (between 5% and 10% of eligible patients).

### Variables

#### Patient Characteristics

Six patient-level variables were included in our analysis: age; sex; socioeconomic deprivation using the Index of Multiple Deprivation (IMD) presented as quintiles^17^; the number of comorbidities according to the Royal College of Surgeons Charlson comorbidity score^18^; residential area classified as rural, urban (outside London), or London^19^; and cancer T stage.

#### Hospital Characteristics

We created 6 hospital-level performance indicators that may make a hospital more attractive to patients and their primary or secondary care physicians when considering where to have surgical treatment. These variables were informed by the peer-reviewed literature,^3^ the National Bowel Cancer Audit’s organizational survey,^20^ and the patient steering committee of the current project.

- Colorectal outcomes: 2-year mortality outcomes are publicly reported for each NHS hospital undertaking major bowel cancer resections by the National Bowel Cancer Audit.^16^ Hospitals were ranked according to their mortality rates for patients diagnosed in 2017 and were subsequently divided into quartiles.
- Overall hospital performance rating: we identified 12 hospitals as providing inadequate care according to the UK Care Quality Commission in 2017. This rating system provides a composite metric for hospital quality across 5 key dimensions of care (safe, effective, caring, responsive, and well-led) and is published online.^21^ Hospitals are graded as outstanding, good, requires improvement, or inadequate.
- Specialist colorectal cancer expertise: we identified 39 specialist colorectal cancer surgery centers. They are designated pelvic exenteration sites where cases requiring specialist colorectal/pelvic surgery input are referred from other hospitals.^20^
- Treatment availability: we identified 51 comprehensive cancer centers. These are hospitals that offer both colorectal surgery and radiotherapy on the same site and all provide neoadjuvant radiotherapy for patients with rectal cancer.
- Robotic surgery: we identified 22 robotic centers. These are the hospitals routinely performing robotic rectal cancer surgeries (at least 10 surgeries per year). No centers were undertaking robotic surgery for colon cancer routinely at the time of analysis.
- Research activity: we defined 31 high-research activity hospitals using an established method based on trial recruitment (eMethods in the Supplement).^22^

#### Travel Time

Patients’ residential locations were represented by the population-weighted centroids of their lower-layer super output areas. There are 32 844 lower-layer super output areas in England defined as small geographic areas that typically include 1500 residents or 650 households.^23^

Travel times were calculated using a geographic information system by inputting the population-weighted centroids of the patients’ lower-layer super output areas and full postcodes of the 163 hospitals providing bowel cancer surgery. The travel time was defined as the fastest route by car (in minutes) using the Ordnance Survey Master Map Highways Network. Travel time was included in the model as the additional travel time patients had to travel beyond their nearest hospital (travel time to the nearest hospital was 0) to reach an alternative hospital providing colorectal cancer surgery.

### Statistical Analyses

#### Hospital Bypassing Model

To assess patient mobility (the extent to which patients receive care at a center other than their nearest), all bowel cancer surgery sites were ranked according to the distance (measured as the mean travel time by car) for each patient. The proportion of patients not receiving care at their nearest bowel cancer surgery center were classified as bypassers were classified as bypassers.

#### Determinants of Treatment Location

We applied conditional logistic regression^4,5^ to estimate the association between where a patient receives surgery and how far it is from the patient’s residence (measured as travel time in minutes), the characteristics of the hospital (according to the 6 hospital-level characteristics defined in the Methods), and the patient’s characteristics (6 patient-level characteristics defined in the Methods).^24^ For each patient, we created a data set that included a row for each of the 163 centers providing colorectal cancer surgery. The outcome was a variable with a value of 1 for the center where the patient had their treatment and a value of 0 otherwise.

We carried out a univariable analysis to assess the association between travel time and each of the 6 hospital characteristics on the odds of patients receiving care in a center at a particular hospital. This was followed by multivariable analyses, including travel time and the hospital characteristics. A third multivariable model included travel time, hospital characteristics, and interaction terms of travel time and patient characteristics. These interactions were included to assess whether the association of travel time with treatment location is modified by patient characteristics. We assessed whether the willingness to travel varies according to age (age ≥70 vs <70 years), comorbidity (0 vs ≥1 comorbidities), socioeconomic status (patients from the least socially deprived areas [IMD score of 1 or 2] vs patients from the most deprived areas [IMD score of 3-5]), sex (male vs female), rural urban classification of patient’s residence (urban vs rural), or cancer T stage (T3/4 vs T1/T2).

Twenty multiple imputations (21.7%) with chained equations were applied to impute the missing values for stage T. Regression results from imputed data sets were combined using Rubin rules. Robust standard errors were estimated to account for the possible clustering of mobility within Cancer Alliances (there are 21 Cancer Alliances across England responsible for coordination of cancer services within their geographical catchment area). All analyses were conducted using Stata version 15 (StataCorp). Two-sided *P* values were statistically significant at .05. Analysis took place between April 2021 and February 2022.

## Results

We identified 46 627 patients who had an elective major bowel resection for colorectal cancer between April 1, 2016, and March 31, 2019, and 44 299 patients (95.0%) were included in the final cohort (eFigure in the Supplement): 31 258 patients with colon cancer and 13 041 patients with rectal cancer. The characteristics of these patients are detailed in Table 1.

**Table 1.** Characteristics of Patients With Colon vs Rectal Cancer Who Underwent a Major Bowel Cancer Resection Between 2016 and 2019 in the English National Health Service

Characteristic	Cancer, No. (%)	
	Colon	Rectal
No.	31 258	13 041
Age, y (at admission)		
<60	5298 (17.0)	3212 (24.6)
60-69	8174 (26.2)	4083 (31.3)
70-79	11 083 (35.5)	4111 (31.5)
≥80	6703 (21.4)	1635 (12.5)
Sex		
Male	16 934 (54.2)	8536 (65.5)
Female	14 324 (45.8)	4505 (34.5)
Socioeconomic deprivation status measured by Index of Multiple Deprivation, quintile		
First (least deprived areas)	7384 (23.6)	2993 (23.0)
Second	7363 (23.6)	2960 (22.7)
Third	6489 (20.8)	2800 (21.5)
Fourth	5551 (17.8)	2375 (18.2)
Fifth (most deprived areas)	4471 (14.3)	1913 (14.7)
Rural/urban classification		
Rural	7154 (22.9)	3134 (24.0)
Urban (non-London)	20 762 (66.4)	8714 (66.8)
London	3342 (10.7)	1193 (9.2)
No. of Charlson Comorbidity Index comorbidities		
0	15 659 (50.1)	7529 (57.7)
1	9765 (31.2)	3701 (28.4)
≥2	5834 (18.7)	1811 (13.9)
Cancer stage, node negative		
T1/T2	5947 (19.0)	2878 (22.1)
T3/T4	6735 (21.6)	2669 (20.5)
Any T, node positive (N1-N3)	11 041 (35.3)	5514 (42.3)
Missing	3501 (11.2)	1331 (10.2)
Hospital characteristics, No. of sites (n = 163)		
Specialist colorectal cancer surgery center (n = 39)	9436 (30.2)	4500 (34.5)
Comprehensive cancer center (n = 51)	12 200 (39.0)	5360 (41.1)
Robotic center (n = 22)	NA	2336 (17.9)
Overall hospital performance rating (No. of hospitals)		
Outstanding (n = 10)	1637 (5.2)	779 (6.0)
Good (n = 47)	9378 (30)	3797 (29.1)
Requires improvement (n = 94)	17 835 (57.1)	7573 (58.1)
Inadequate (n = 12)	2408 (7.7)	892 (6.8)
Research activity (No. of hospitals), quintiles		
First-fourth (n = 132)	25 193 (80.6)	10 270 (78.8)
Fifth highest (n = 31)	6065 (19.4)	2771 (21.3)
Adjusted 2-y mortality, quartile (No. of centers)		
First (lowest) (n = 41)	7161 (22.9)	3007 (23.1)
Second (n = 40)	7933 (25.4)	3362 (25.8)
Third (n = 41)	8254 (26.4)	3424 (26.3)
Fourth (highest) (n = 40)	7766 (24.8)	3188 (24.5)
Missing (1)	144 (0.5)	60 (0.5)

Abbreviation: NA, not applicable.

### Hospital Bypassing

Overall, 8550 of 31 258 patients with colon cancer (27.4%) and 3933 of 13 041 patients with rectal cancer (30.2%) bypassed their nearest hospital providing bowel cancer surgery (mean [SD] age, 68.9 [11.6] years; 18 829 [42.5%] female). The proportion was higher in patients living in rural areas (2114 of 7154 [29.5%] for colon cancer and 1052 of 3134 [33.6%] for rectal cancer) compared with patients living in urban non-London areas (4967 of 20 762 [23.9%] for colon cancer and 2321 of 8714 [26.6%] for rectal cancer). The Figure shows the area of residence for patients who had rectal cancer surgery at a selected surgery center in London. This included patients who lived within the local area of the center as well patients who traveled from outside of the local area to receive care there (bypassers). Table 2 highlights the median travel time for nonbypassers and bypassers according to the number of hospitals bypassed.

**Figure.**
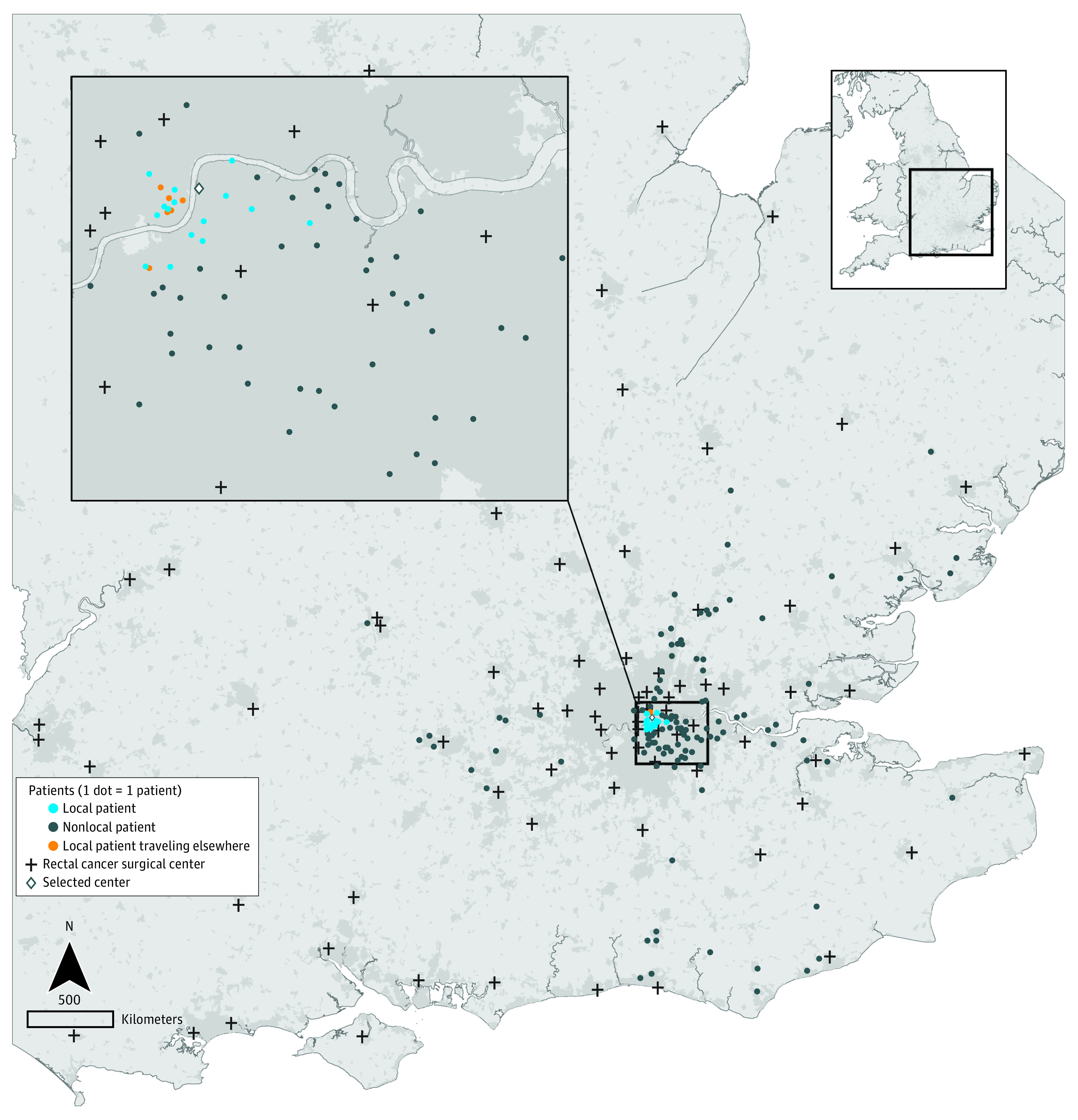
Mobility Patterns of Patients Receiving Radical Rectal Cancer Surgery at a Selected English National Health Service Surgery Center Map of the Southeast region of England (UK), illustrating the mobility patterns of patients who received radical rectal cancer surgery at a selected National Health Service surgery center located in London indicated with a diamond symbol in the area of local users (light blue). Patients treated at the center who traveled from outside the local area (bypassers) are represented as dark blue dots. Patients from the center’s local area who traveled to other centers for surgery are represented as orange dots. The map includes a scaled magnification of the region inset and a national overview. Contains National Statistics and National Records of Scotland data (source: Northern Ireland Statistics and Research Agency) as well as Ordnance Survey data. ©Crown copyright and database right 2022.

**Table 2.** Patient Mobility of Patients With Colon vs Rectal Cancer Who Underwent a Major Bowel Cancer Resection Between 2016 and 2019 in the English National Health Service

No. of hospitals bypassed	Patients, No. (%)	Travel time by car, median (IQR), min
**Colon cancer (n = 31 258)**		
0	22 708 (72.7)	11.2 (7.0-19.1)
1	4326 (13.8)	18.8 (12.3-29.6)
2	1491 (4.8)	21.6 (14.3-33.2)
3	947 (3.0)	26.9 (15.8-38.2)
4	381 (1.2)	24.6 (16.6-43.6)
≥5	1405 (4.5)	33.6 (25.1-61.6)
**Rectal cancer (n = 13 041) **		
0	9108 (69.8)	11.2 (7.1-18.9)
1	1833 (14.1)	20.6 (13.2-32.6)
2	655 (5.0)	24.4 (14.7-35.7)
3	404 (3.1)	26.0 (16.0-36.7)
4	228 (1.8)	28.3 (18.7-4316)
≥5	813 (6.2)	40.1 (26.9-67.1)

### Association of Travel Time, Hospital, and Patient Characteristics With Treatment Location

For both colon and rectal cancer surgery, the univariable and multivariable analysis (Table 3) demonstrated that travel time was strongly associated with where the patient received their surgery. The odds of a patient traveling to another surgery center than the nearest rapidly decreased with the additional travel time. For example, the odds of patients with colon cancer traveling to a center that was up to 10 minutes further away than their nearest surgery center was considerably lower (odds ratio [OR], 0.24 in the multivariable model), which is in keeping with most patients receiving care at their nearest center.

**Table 3.** Univariable and Multivariable Analyses Estimating the Association Between Travel Time and Hospital Characteristics on Treatment Location in Patients With Colon vs Rectal Cancer

Characteristic	Univariable analysis (95% CI)^a^	*P* value	Multivariable analysis (95% CI)^b^	*P* value
**Colon cancer (n = 31 258)**				
Additional travel time, min				
0 (Nearest hospital)	1 [Reference]	<.001	1 [Reference]	<.001
1-10	0.23 (0.18-0.28)^c^		0.22 (0.18-0.28)^c^	
11-30	0.01 (0.01-0.02)^c^		0.01 (0.01-0.02)^c^	
31-60	0.001 (0.0009-0.002)^c^		0.001 (0.0008-0.002)^c^	
>60	0.00008 (0.00005-0.0001)^c^		0.00008 (0.00005-0.0001)^c^	
Hospital characteristics				
Specialist colorectal cancer surgery center	1.38 (0.99-1.92)	.06	1.16 (0.87-1.56)	.32
Comprehensive cancer center	1.41 (0.98-2.02)	.06	1.14 (0.84-1.54)	.40
Robotic center				
Overall hospital performance rating: inadequate	1.05 (0.59-1.88)	.87	0.83 (0.62-1.11)	.20
Research activity: highest 20%	1.03 (0.59-1.79)	.93	0.90 (0.67-1.21)	.48
Adjusted 2-y mortality, quartile				
First (lowest)	1 [Reference]	.92	1 [Reference]	.07
Second	1.14 (0.82-1.57)		1.37 (1.09-1.72)	
Third	1.15 (0.79-1.71)		1.54 (1.09-2.19)	
Fourth (highest)	1.11 (0.72-1.71)		1.08 (0.85-1.37)	
Missing	0.82 (0.13-5.35)		1.32 (0.32-5.43)	
**Rectal cancer (n = 13 041) **				
Additional travel time, min				
0 (Nearest hospital)	1 [Reference]	<.001	1 [Reference]	<.001
1-10	0.25 (0.20-0.32)^c^		0.24 (0.19-0.30)^c^	
11-30	0.02 (0.01-0.02)^c^		0.02 (0.01-0.02)^c^	
31-60	0.002 (0.002-0.003)^c^		0.002 (0.001-0.003)^c^	
>60	0.0001 (0.0001-0.0002)^c^		0.0001 (0.0001- 0.0002)^c^	
Hospital characteristics				
Specialist colorectal cancer surgery center	1.68 (1.24-2.26)^c^	.001	1.45 (1.13-1.87)^c^	.004
Comprehensive cancer center	1.53 (1.07-2.19)	.02	1.14 (0.87-1.49)	.33
Robotic center	1.40 (0.96-2.03)	.08	1.43 (1.11-1.86)^c^	.007
Overall hospital performance rating: inadequate	0.92 (0.51-1.68)	.80	0.70 (0.50-0.97)^c^	.03
Research activity: highest 20%	1.15 (0.67-1.97)	.61	1.17 (0.90-1.51)	.25
Adjusted 2-y mortality, quartile				
First (lowest)	1 [Reference]	.92	1 [Reference]	.08
Second	1.15 (0.83-1.58)		1.28 (1.05-1.56)	
Third	1.14 (0.75-1.74)		1.45 (1.04-2.01)	
Fourth (highest)	1.09 (0.72-1.63)		1.03 (0.82-1.30)	
Missing	0.82 (0.12-5.58)		1.10 (0.24-5.08)	

^a^ Model 1 presents unadjusted odds ratio from the univariable conditional logit analysis assessing the association of additional travel time and each hospital characteristic with the odds that a patient travels to a particular hospital. *P* values are based on likelihood ratio test.

^b^ Model 2 presents adjusted odds ratio from the multivariable conditional logit analysis assessing the association of additional travel time and hospital characteristics with the odds that a patient travels to a particular hospital. *P* values are based on likelihood ratio test.

^c^ Statistically significant.

For colon cancer, there were no statistically significant associations in the multivariable model between any of the hospital characteristics and the odds of a patient traveling to a particular hospital (Table 3). For rectal cancer, we found that patients were more likely to travel to a hospital that is a designated complex colorectal cancer surgery center (OR, 1.45; 95% CI, 1.13-1.87; *P* = .004) and to a hospital performing robotic surgery for rectal cancer (OR, 1.43; 95% CI, 1.11-1.86; *P* = .007). In addition, patients were less likely to travel to hospitals deemed to have inadequate care according to the overall hospital performance rating (OR, 0.70; 95% CI, 0.50-0.97; *P* = .03). For both colon and rectal cancer, we did not find any association between the odds of patients traveling to a particular hospital and the 2-year bowel cancer mortality outcomes.

The interaction terms presented in Table 4 establish the variation in the association between travel time and treatment location according to 6 patient characteristics. We found that the association between travel time and treatment location was stronger for older patients, and for more patients with low socioeconomic status. It was less strong for patients living in rural areas and for patients with more advanced cancer stage. For example, additional travel time was associated with reduced odds of a patient 70 years or older traveling to an alternative more distant hospital to a greater extent (OR for the interaction term always <1) compared with patients younger than 70 years. In other words, patients 70 years and older have a lower willingness to travel. Conversely, additional travel time was less strongly associated with the odds of traveling to a particular hospital for patients who lived in rural areas (OR for interaction term always >1) compared with patients living in urban areas, ie, patients living in rural areas had a greater willingness to travel.

**Table 4.** Multivariable Analysis Estimating the Association of Travel Time and Patient and Hospital Characteristics With Treatment Location in Patients With Colon vs Rectal Cancer^a^

Characteristic	Cancer			
	Colon (n = 31 258)		Rectal (n = 13 041)	
	Adjusted OR (95% CI)	*P* value	Adjusted OR (95% CI)	*P* value
Additional travel time, min^b^				
0 (Nearest hospital)	1 [Reference]	<.001	1 [Reference]	<.001
1-10	0.21 (0.17-0.26)^c^		0.21 (0.16-0.27)^c^	
11-30	0.01 (0.01-0.02)^c^		0.02 (0.01-0.02)^c^	
31-60	0.001 (0.0006-0.003)^c^		0.002 (0.001-0.003)^c^	
>60	0.00008 (0.00004-0.0002)^c^		0.0001 (0.00008-0.0002)^c^	
**Travel time by patient characteristics, min^d^**				
Age ≥70 y vs <70 y				
1-10	0.97 (0.91-1.04)^c^	<.001	0.98 (0.86-1.10)^c^	<.001
11-30	0.77 (0.67-0.88)^c^		0.78 (0.67-0.91)^c^	
31-60	0.58 (0.42-0.80)^c^		0.65 (0.51-0.84)^c^	
>60	0.41 (0.28-0.60)^c^		0.45 (0.39-0.59)^c^	
Female vs male				
1-10	1.00 (0.93-1.09)	.40	0.98 (0.89-1.08)	.57
11-30	1.03 (0.93-1.14)		0.97 (0.77-1.22)	
31-60	0.91 (0.73-1.13)		0.97 (0.77-1.23)	
>60	1.23 (0.88-1.71)		1.14 (0.95-1.37)	
Areas with lower levels of socioeconomic vs higher levels, min				
1-10	1.27 (1.09-1.48)^c^	.001	1.27 (1.06-1.51)^c^	.003
11-30	1.06 (0.91-1.23)^c^		1.07 (0.89-1.28)^c^	
31-60	1.14 (0.77-1.69)^c^		1.00 (0.70-1.43)^c^	
>60	0.75 (0.51-1.12)^c^		0.82 (0.59-1.14)^c^	
Rural vs urban areas				
1-10	1.79 (1.46-2.20)^c^	<.001	1.82 (1.43-2.31)^c^	<.001
11-30	2.94 (2.33-3.70)^c^		2.76 (2.17-3.52)^c^	
31-60	2.38 (1.64-3.45)^c^		2.10 (1.34-3.30)^c^	
>60	2.24 (1.66-3.01)^c^		1.95 (1.35-2.80)^c^	
≥1 Comorbidity vs no comorbidity				
1-10	0.93 (0.84-1.02)	.08	0.92 (0.84-1.01)	.23
11-30	0.94 (0.83-1.06)		0.94 (0.84-1.05)	
31-60	1.11 (0.80-1.53)		1.08 (0.84-1.38)	
>60	0.79 (0.56-1.10)		0.97 (0.73-1.30)	
Stage T3/4 disease vs stage T1/2 disease, min				
1-10	0.96 (0.89-1.04)	.17	1.06 (0.93-1.21)^c^	.04
11-30	0.97 (0.85-1.11)		1.04 (0.85-1.28)^c^	
31-60	1.10 (0.87-1.38)		1.29 (0.97-1.73)^c^	
>60	1.41 (1.00-1.97)		1.42 (0.97-2.08)^c^	
**Hospital characteristics**				
Specialist colorectal cancer surgery center	1.13 (0.86-1.48)	.38	1.42 (1.12-1.79)^c^	.004
Comprehensive cancer center	1.15 (0.87-1.53)	.33	1.16 (0.90-1.49)	.26
Robotic center	NA	NA	1.40 (1.09-1.81)^c^	.01
Overall QC hospital performance rating: inadequate	0.82 (0.62-1.10)	.18	0.70 (0.50-0.97)^c^	.03
Research activity: highest 20%	0.90 (0.66-1.23)	.51	1.16 (0.89-1.52)	.27
Adjusted 2-y mortality, quartile				
First (lowest)	1 [Reference]	.06	1 [Reference]	.06
Second	1.34 (1.07-1.68)		1.25 (1.04-1.50)	
Third	1.50 (1.08-2.09)		1.42 (1.05-1.92)	
Fourth (highest)	1.04 (0.81-1.34)		0.99 (0.78-1.25)	
Missing	1.31 (0.35-4.97)		1.12 (0.26-4.87)	

Abbreviations: NA, not applicable; OR, odds ratio; QC, quality control.

^a^ Model 3 presents adjusted ORs from the multivariable conditional logit analysis assessing the association of additional travel time, hospital characteristics, and patient characteristics on the odds that a patient travels to a particular hospital. *P* values are based on likelihood ratio test.

^b^ Note that the adjusted ORs for the association of additional travel time with treatment location in model 3 for colon or rectal cancer relates to a particular patient group: male individuals, age <70 years, without comorbidities, with localized T1/T2 disease, living in urban areas of higher deprivation (Index of Multiple Deprivation 3-5).

^c^ Statistically significant.

^d^ The association of patient characteristics on additional travel time is presented as interaction terms. An OR <1 for the interaction term indicates a stronger association of travel time on the odds of a patient traveling to a particular hospital for that patient group and conversely an OR for the interaction term >1 indicates a weaker association.

## Discussion

In this national population study, we demonstrate that approximately 3 of 10 patients with colorectal cancer who undergo a major resection bypass their nearest bowel cancer surgical center for their treatment. We found that travel time is the most important determinant of where patients receive their treatment. However, patients who were younger, more affluent, or those living in rural areas were more likely to travel to more distant hospitals for surgery. Patients with rectal cancer, irrespective of travel time, were more likely to travel to hospitals routinely offering robotic surgery or those designated as specialist colorectal surgery centers. They were also less likely to travel to hospitals given an inadequate overall performance rating by the national care quality regulator in England. However, we did not find any association between the odds of patients traveling to hospitals with better published 2-year bowel cancer mortality outcomes. For colon cancer, none of the quality measures was associated with choice of treatment location.

Our findings have several policy implications. First, the variation in mobility patterns between patients with rectal and colon cancer may reflect differences in the management pathways of rectal surgery compared with colon cancer surgery.^25,26,27,28^ Patients with rectal cancer may have a greater opportunity for discussion and review of options in their management pathway compared with colon cancer given they could require chemoradiation, undergo a period of surveillance, or be told upfront that they may require a stoma, which many would be keen to avoid. In addition, the technical complexity of rectal cancer surgery compared with colon cancer surgery could prompt patients to consider more carefully their treating surgeon or center. It is important to note that it is likely that the hospital where patients chose to have their treatment also reflects the advice they received from their primary or secondary care physicians.

Second, the effect of patient choice policies on equity remains a key concern, given that older patients and patients from poorer socioeconomic backgrounds were less likely to receive care at a center other than their nearest. This has been observed in other studies assessing the association of patient characteristics with the choice of treating hospital.^3^ We also found that patients with more advanced rectal cancers were more likely to travel for surgical treatment. If younger and more affluent patients are prepared to travel further, this could enhance inequities in outcomes of older, more medically and socially complex patient groups.

Third, we demonstrate that even in health care systems that publicly report procedure-specific outcome measures, the availability of robotic surgery was strongly associated with patterns of patient choice and mobility.^29^ Robotic surgery has become one of the most significant technological markers of reputation in health care systems in different cancer types and has been shown to influence patterns of mobility,^3,4,7,30,31^ despite limited evidence to support its routine use.^10,32,33^ In the US health care system, use of robotic surgery for colon cancer surgery increased from 1.8% in 2012 to 15.1% in 2018.^12^ Without more stringent oversight, the unproven benefits of these technologies in some tumor types and increased costs risks increasing inefficiency in cancer care systems.^34^

Fourth, indicators of overall round hospital performance were strongly associated with where patients had their treatment. Therefore, in health care markets where health care funding follows the patient, public reporting of hospital performance could be used as a driver of improvements in clinical quality as hospitals try to maintain their market share and reduce financial losses.^35^

Fifth, patients did not seem to be sensitive to colorectal cancer surgery quality, despite publicly reporting hospital-level mortality rates.^14,36^ We chose mortality rates rather than readmission rates or permanent stoma rates because we hypothesized these would be easier for patients to understand as a quality measure. One potential reason for this finding is that the awareness among patients that these measures exist is typically poor.^37^ Further engagement work is required to ensure that patients and their families are aware of this information and to understand better the choice architecture across the system.

Sixth is the notion of observable quality or expertise in cancer management.^6^ We found that patients were more likely to travel for treatment at specialized colorectal centers where complex colorectal procedures are performed (eg, pelvic exenteration for primary or recurrent disease). This may be linked to a perception that patient outcomes at hospitals with highly specialized colorectal surgery services is better.

### Limitations

Our modeling of patient mobility does highlight a number of conceptual and methodological challenges. In this article, we have studied where patients had their treatment in relation to where they live. However, decisions are made by patients together with primary or secondary care physicians in the context of preexisting referral patterns. Therefore, it is not possible to say explicitly to what extent these choices reflect the preferences of patients or physicians.^6,38^

The point of referral to an alternative more distant center could have been made for the initial diagnostic investigation or following a colon or rectal cancer diagnosis. We did not have information on the time point of referral, or whether a primary or secondary care physician initiated the referral. We have previously undertaken qualitative work to understand the pathway of referral for prostate cancer,^6^ which showed that organizational factors, including the availability of alternative centers within reasonable travel distance had a major impact on where patients had their treatment. In addition, the reputation and the availability of advanced technologies were associated with perceived quality of care. Qualitative study involving patients with colorectal cancer and primary care physicians is planned. Of note, the observed associations may reflect other unobserved elements of hospital quality, particularly for colon cancer, and we cannot definitively say whether the quality measures presented were the reason for referral to these centers.

The study used centroids of small geographical areas (typically representing 650 households) to represent the location of the patients’ residence and therefore this could have suppressed variation in travel times, attenuating rather than enhancing the observed associations.^39^ In addition, we used an administrative data set, and therefore, we may have missed other potential determinants of mobility such as caregiver or work location.

Finally, we did not include hospital-procedure volume or hospital waiting time as a quality measure into our estimations because of the issue of reverse causality. In theory, patients would prefer to undergo procedures in hospitals delivering high-quality care or in hospitals that would offer them more timely treatment. However, a high-volume hospital may also be high volume because of patient mobility patterns, which would also contribute to the size of the waiting lists of the treating hospital.

## Conclusions

In this national population-based study, we found that up to 30% of patients with colorectal cancer receive surgery at a surgical center other than their nearest. Patients with rectal cancers were responsive to measures of overall hospital quality but less so to publicly reported specific disease-related outcome measures. Despite publication of hospital-level outcomes, hospitals routinely offering robotic surgery for rectal cancer were more likely to attract patients from the catchment areas of other hospitals. The study also demonstrates that younger, fitter, more affluent patients were more likely to travel to alternative hospitals for surgery. This highlights that patient choice policies may drive inequities in the health care system if specific patient groups are able to access better quality care.

## Notes

Supplement. eMethods. eFigure. Flow diagram of patients included in the study
