# Supplementary material for: Association of Quality and Technology With Patient Mobility for Colorectal Cancer Surgery
Source: JAMA Surg. 2022 Nov 9;158(1):e225461. doi: 10.1001/jamasurg.2022.5461 (PMC9647575; doi:10.1001/jamasurg.2022.5461)
Supplement: Supplement. — eMethods. eFigure. Flow diagram of patients included in the study [file jamasurg-e225461-s001.pdf]

Supplemental Online Content

Aggarwal A, Han L, Boyle J, et al. Association of quality and technology with patient mobility for colorectal cancer surgery. *JAMA Surg*. Published online November 9, 2022. doi:10.1001/jamasurg.2022.5461

eMethods.

eFigure. Flow diagram of patients included in the study

This supplemental material has been provided by the authors to give readers additional information about their work.

© 2022 Aggarwal A et al. *JAMA Surgery.*

eMethods

Development of hospital performance metric based on research activity

- Research activity – we defined 31 'high-research activity' hospitals using an established method based on trial recruitment (e-Methods in the Appendix) [23] that considered research activity at a hospital according to the number of participants recruited at each hospital per annum to federally funded (NHS National Institute for Health Research) Clinical Research Network Studies (n=approx. 6000 studies) in 2018/19. We normalised the recruitment numbers according to hospital size by using overnight bed capacity as a proxy. The metric for research activity created was then divided into quintiles.

© 2022 Aggarwal A et al. *JAMA Surgery.*

eFigure. Flow diagram of patients included in the study

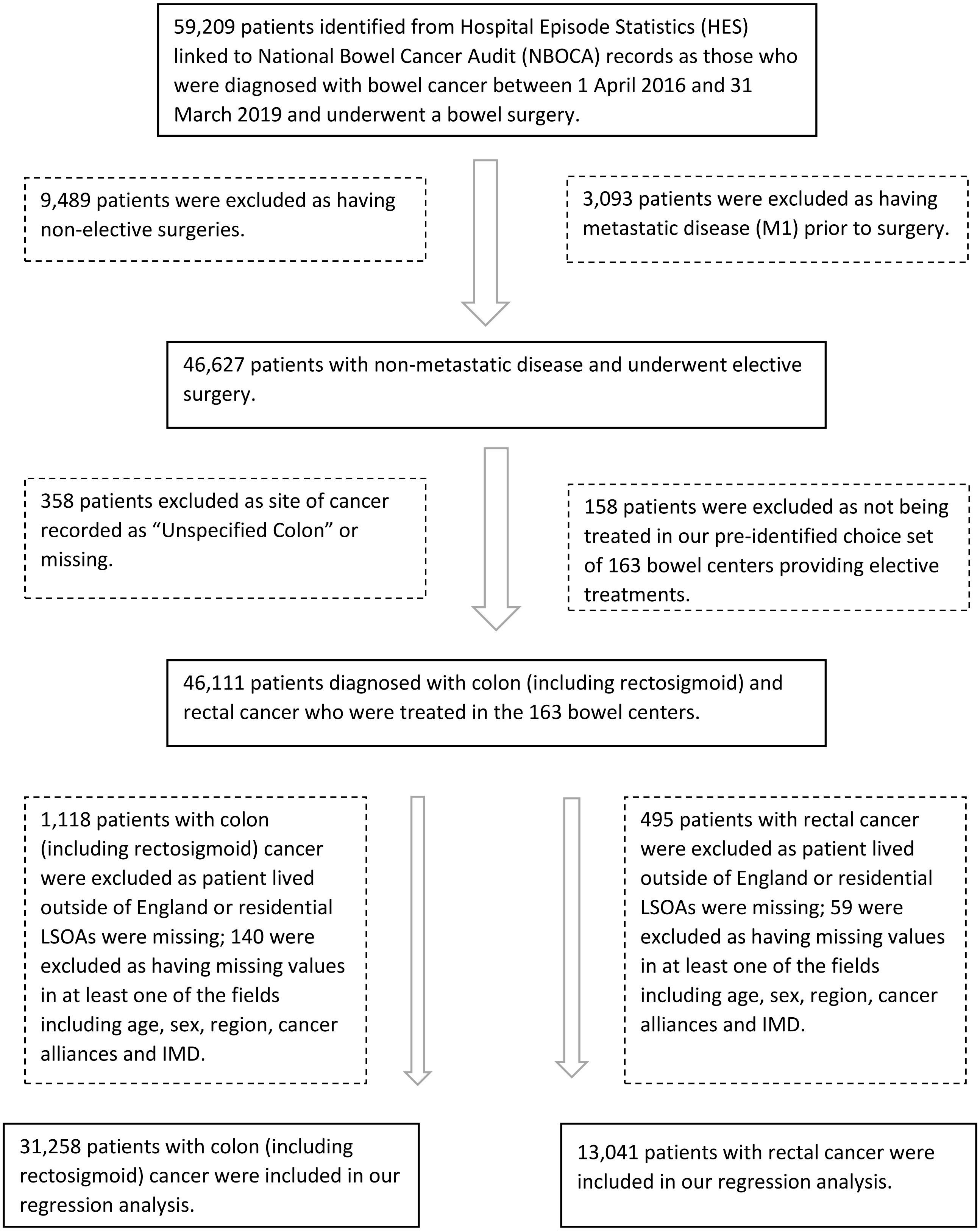

© 2022 Aggarwal A et al. *JAMA Surgery.*
